# A Novel Computer Oculomotor Rehabilitation (COR) Program for Mild Traumatic Brain Injury (mTBI)

**DOI:** 10.3390/brainsci7080099

**Published:** 2017-08-09

**Authors:** Kenneth J. Ciuffreda, Naveen K. Yadav, Preethi Thiagarajan, Diana P. Ludlam

**Affiliations:** 1 Department of Biological and Vision Sciences, State University of New York, College of Optometry, New York, NY 10016, USA; preethi.thiagarajan@gmail.com (P.T.); dianaeye@aol.com (D.P.L.); 2 Chicago College of Optometry, Midwestern University, Downers Grove, IL 60515, USA; nyadav@midwestern.edu

**Keywords:** eye movements, traumatic brain injury (TBI), vision therapy, vision rehabilitation, reading, visual scanning, perceptual and motor learning, neuroplasticity, computer-based therapy

## Abstract

Individuals with traumatic brain injury (TBI) manifest a wide range of visual dysfunctions. One of the most prevalent involves the oculomotor system, which includes version, vergence, and accommodation. However, until recently, there has been no comprehensive, computer-based program for remediation of these oculomotor deficits. We present such an oculomotor rehabilitation program that has been tested in a clinical trial in patients having TBI with a high degree of success based on before-and-after objective system recordings, performance measures, and related visual symptomotology. The basic program components include a versatile stimulus package incorporating the attentional paradigm of rapid serial visual presentation (RSVP), the ability to add a visual and/or auditory distractor to the training to increase difficulty level (“task loading”), automated assessment of RSVP errors, and automated assessment of visual performance over the training period. Program limitations and future directions are also considered.

## 1. Introduction

Individuals afflicted with a traumatic brain injury (TBI) may experience a wide range of dysfunctions of a sensory, motor, perceptual, cognitive, attentional, behavioral, pharmacologic, somatic, and/or linguistic nature [1,2] due to the pervasiveness of the injury (e.g., coup-contrecoup). For example, the patient may have difficulty sleeping, manifest cognitive and attentional deficits, exhibit inappropriate behaviors, and have gait and postural biases and maladaptations. However, there are well-accepted diagnostic and therapeutic means to assist these individuals [1,2].

Similarly, individuals with TBI frequently exhibit a constellation of vision and visual information processing dysfunctions with an oculomotor and/or non-oculomotor basis [3,4]. This is not surprising, as the majority of the 12 cranial nerves and at least 30–40 areas of the brain are involved in the visual process [4]. For example, the patient may have a visual field defect, exhibit photosensitivity and visual motion sensitivity, manifest visual-attentional problems, and evidence oculomotor deficits [3,4]. Still, as mentioned above for the general dysfunctions, there are improved and evolving diagnostic and therapeutic means to assist these individuals with their vision problems [5,6].

One of the most common vision problems reported by TBI patients, in particular those with the more mild form (mTBI), is an oculomotor/eye movement deficit with its adverse effects on general visual scanning/visual search and reading [7,8,9,10,11,12,13,14,15,16,17,18,19,20]. Again, multiple areas of the brain are involved in eye movement control and reading [21,22]. Over the past several decades, there have been efforts to develop clinical and laboratory-based methods for the training of eye movements and their relation to general vision and reading-related visual activities [23,24,25,26]. Only relatively recently have they been targeted for the TBI population including SHAM (an inactive treatment or procedure that is intended to mimic as closely as possible a therapy in a clinical trial) control [26].

However, efforts to design eye movement training programs have been relatively modest and non-comprehensive in nature, that is with restricted goals in mind (e.g., targeting only the versional eye movement system) [25]. Thus, the purpose of the present paper is to present the conceptual framework for a more comprehensive and functional training program, specifically with the mTBI/TBI patient in mind. In fact, much of the proposed training program has been tested by the authors in a DoD (Department of Defense) sponsored clinical trial in the mTBI population, with considerable success [14,15,16,17,18,19,20].

## 2. General Program Concepts

**Figure 1:**
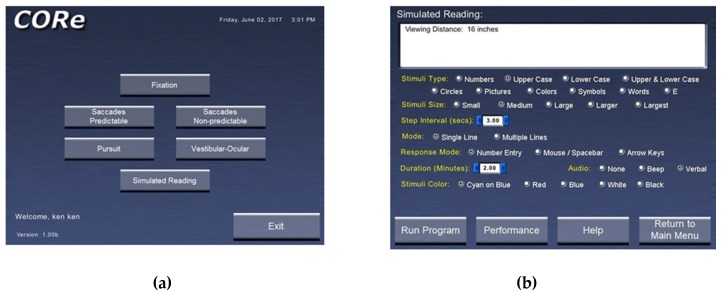
(**a**) CORe program home page (**b**) Sample interface screen of CORe program for simulated reading training [17,26].

There are several key components to the computer oculomotor rehabilitation (COR) program approach. In addition to the basic introductory aspects, and brief patient information input, there is:

1. A window displaying the possible categories of eye movement training. These currently include fixation in different positions of gaze (central, horizontal, and vertical); predictable saccades (horizontal and vertical); non-predictable saccades (horizontal and vertical); smooth pursuit (horizontal and vertical): vestibulo-ocular reflex in different positions of gaze (central, horizontal, vertical, and oblique); and simulated reading (Figure 1a). Figure 1b shows the specific template for training reading eye movements.
2. Related to #1, there is the ability to vary the stimulus parameters for each category of eye movement trained. For example, for smooth pursuit training, direction (horizontal or vertical), amplitude (5 or 10 degrees), target velocity (0.5–10.0 degrees per second), and size (in five increments ranging from very small to very large), as well as other parameters (e.g., color), can be varied to maintain attention and/or gradually increase the level of task difficulty (i.e., task loading).
3. Related to #1 and 2, the concept of rapid serial visual presentation (RSVP) is employed [27]. RSVP represents an experimental approach to assess temporal aspects of general/visual attention. RSVP involves the use of a “targeted” stimulus presented immediately prior to the training trial, as well as various, random “distractor” stimuli that are presented along with the “targeted” stimulus during a trial run. For example, as the patient trains fixation on the midline for 30 s, either the targeted or distractor stimulus is presented multiple times. The task is to perform the oculomotor training while concurrently counting how many times the “targeted” stimulus was presented with respect to the total number of stimulus changes that occurred (e.g., four times out of 12). The RSVP paradigm forces an additional attentional aspect to the training task. RSVP is incorporated into all of the training programs.
4. The ability to add a visual and/or auditory distractor during training is possible. The visual distractor is comprised of a white, rectangular bar that is spatially and temporally presented at random, concurrently with the training stimulus during a trial, if so desired. Similarly, the audio distractor is comprised of “white noise” that is present throughout the trial, if so desired. This feature introduces increased “task-loading” to the training, which adds an additional layer of difficulty, once the patient begins to perform better. It is also possible to have neither distractor, if so desired, especially at the early training sessions when the patient’s performance is less than optimal.
5. There is basic monitoring of progress for each category of training over time. This feature provides the number of “targeted” stimuli and the number of errors for each training trial, and then for all trials performed at a particular session, as well as across sessions on different days/weeks/months.
6. Lastly, one can manually introduce plus or minus spherical lenses to alter the accommodative (eye focusing) near demand, as well as either base-out or base-in prisms to alter the horizontal vergence near demand, or both. Base-up and base-down prisms can also be introduced to train the vertical vergence system. Thus, all three basic oculomotor control systems can be activated during a training session, namely, vergence, accommodation, and version.

### Specific Program Concepts

All categories of eye movements have the following choices:

1. Stimulus type: There are 10 possible choices—a number, upper case letter, lower case letter, upper and lower case letters combined, colored balls, symbols, short words, and a Snellen E.
2. Stimulus size: There are five possible choices—small, medium, large, larger, and largest (2–20 mm in dimension).
3. Duration per position: 0.25–5.0 min.
4. Response mode: number entry, mouse/spacebar entry, or arrow key entry for the patient to indicate the number of targeted stimuli they believed to be present during a trial run.
5. Stimulus color: cyan on blue, blue, red, white on black, and black on white.
6. Distractor: none, visual, audio, or both.
7. Audio performance indicator: none, beep, or computer-based human spoken word to indicate if the number of RSVP targeted changes entered was correct.
8. Rapid serial visual presentation (RSVP).

Additional options for each specific category of eye movements are as follows:

1. Fixation:
  - presentation time on (0.1–4 s)
  - presentation time off (0–4.0 s, with zero indicating no time gap between stimulus presentation)
  - stimulus placement—all five possible positions, namely either center, or right, left, top, or down (all 10 degrees from center)
2. Predictable saccades:
  - step interval change (1–10 s)
  - stimulus amplitude (5 or 10 degrees centered on the midline)
  - direction of stimulus movement (horizontal or vertical)
3. Non-predictable saccades
  - does not have 2a and 2b above
  - trial duration (0.25–5.0 min)
  - direction of stimulus movement (horizontal or vertical centered on the midline)
4. Smooth Pursuit
  - velocity (0.5–10 degs/s)
  - direction of stimulus movement (horizontal or vertical centered on the midline)
5. Vestibulo-ocular reflex (VOR)
  - stimulus placement—either center, or left, right, up, down, upper left, upper right, lower left, and lower right (all 10 degrees from center)
  - metronome beat frequency—0.25–4.0 beats/s—indicates head movement frequency per cycle while fixating and maintaining stability of the target; up to 6 Hz if possible.

## 3. Examples of Training Benefits

Across the range of studies over the past nearly two decades [7,8,9,10,11,12,13,14,15,16,17,18,19,20], and even decades earlier [28], there have been a multitude of examples showing the positive effects of vision therapy on basic oculomotor control, as well as reading, in the mTBI population using objectively-based recording techniques and detailed quantification. Some examples are provided below in three different patients from our recent studies.

**Figure 2:**
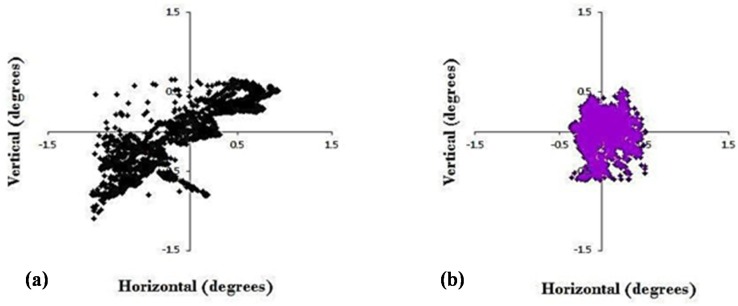
Two-dimensional plot of binocular central fixation before (**a**) and after (**b**) oculomotor training in a typical mTBI subject. Data presented from the right eye [17].

The first shows two-dimensional, horizontal, and vertical eye position over a 30-s period of binocular fixation at near before and after 9 h total of vision therapy in an adult with chronic mTBI [17] (Figure 2). The 9 h were imposed by the constraints of the clinical trial design, which included 3 h each of version, vergence, and accommodative therapy (Table 1). The dispersion of values was approximately ±1.0 degree in all directions prior to the therapy. In contrast, after the brief period of therapy, the overall dispersion reduced by approximately 50% (±0.5 degree), which was statistically significant (*p* < 0.05). Normal values are about ±0.25 degrees [22]. The presumption is that with more hours of therapy (e.g., 20–30 h total), which is the typical clinical scenario in these patients, the improvement would be even better, but this awaits another clinical trial to test a dose-based hypothesis.

**Figure 3:**
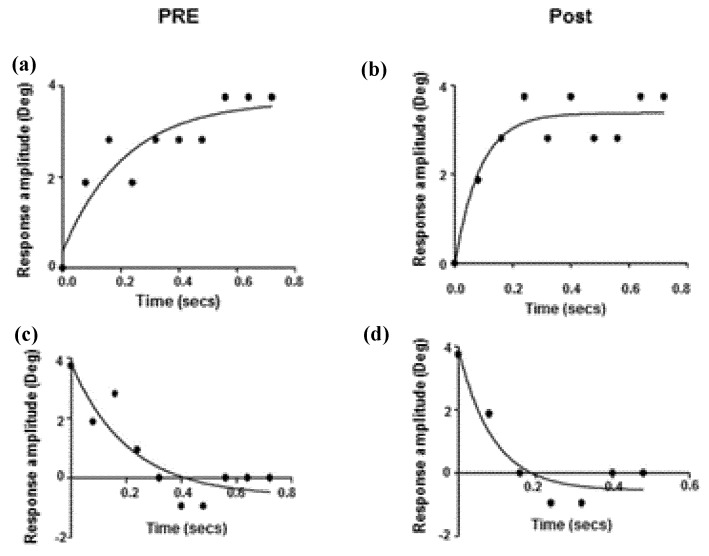
Horizontal vergence eye position as a function of time. Exponential fit of step vergence dynamic trajectory from right eye before (**a,c**) and after (**b,d**) oculomotor training for convergence (**a,b**) and divergence (**c,d**) in a typical mTBI subject [14].

The second shows binocular, horizontal, vergence eye position as a function of time before and after the 9 h of therapy (per the above description; Table 1) [14] (Figure 3). For both convergence and divergence, the time to reach the final, steady-state, binocular eye position reduced (i.e., became quicker) by approximately 50% following the prescribed period of vision therapy. Similarly, and as expected, the correlated vergence response peak velocity increased by about 50%, after the therapy. While these two parameters did not normalize, the changes were significantly different (*p* < 0.05), thus showing a large and positive therapeutic effect. Again, additional therapy might have resulted in even more improvement.

**Figure 4:**
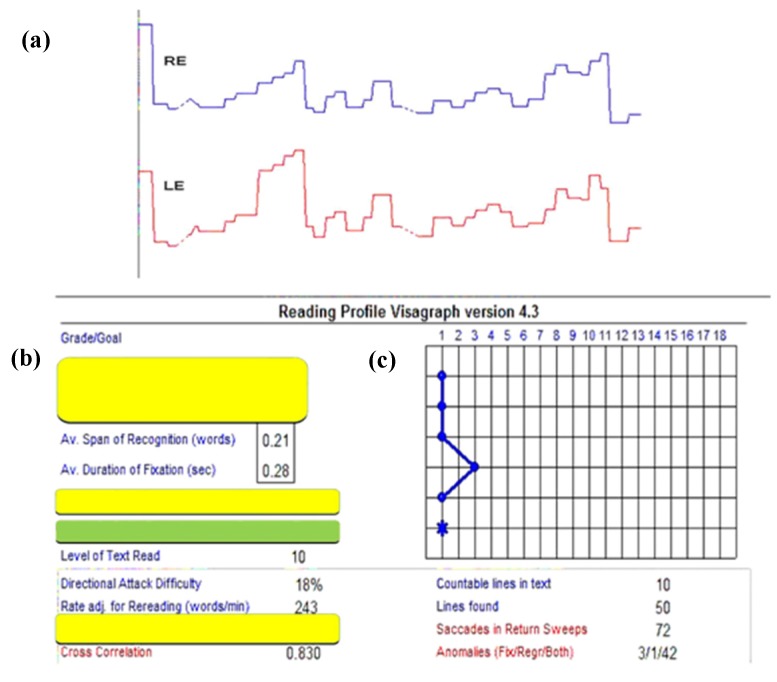
Visagraph output in a typical mTBI subject at baseline: (**a**)—Horizontal eye position as a function of time (8 s data); RE = right eye; LE = left eye; Upward inflection = progressive saccade; downward inflection = regressive saccade. (**b,c**)—On the left (**b**) = Various Visagraph parameters assessed for grade-10 reading material; Graph plot on the right (**c**) = Taylor’s [24] grade level efficiency (from 1–18; >12 is normal), showing a level of 1.0 (blue star) in this subject. Relevant parameters are highlighted [15].

**Figure 5:**
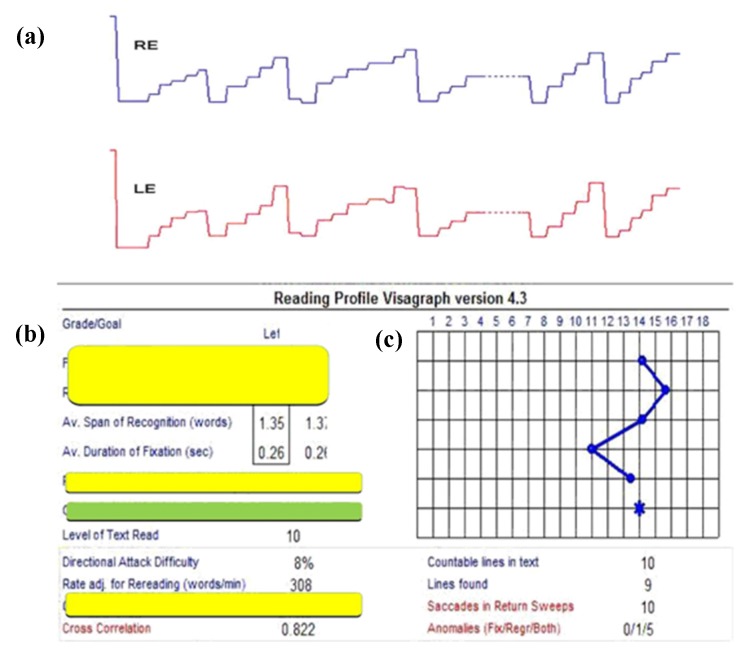
Visagraph output in a typical mTBI subject after oculomotor training: (**a**)—Horizontal eye position as a function of time (8 s data); RE = right eye; LE = left eye; Upward inflection = progressive saccade; downward inflection = regressive saccade. (**b,c**)—On the left (**b**) = Various Visagraph parameters assessed for grade-10 reading material; Graph plot on the right (**c**) = Taylor’s [24] grade level efficiency (from 1–18; >12 is normal, based on weighted average of the reading parameters), showing a level of 14.0 (blue star) in this subject. Relevant parameters are highlighted [15].

Lastly, the third example shows the binocular versional, horizontal reading eye movements as a function of time after the same 9 h of vision therapy [15] (Table 1) (Figure 4 and Figure 5). Following therapy, the reading eye movements became more regular and "step-like", similar to that expected in a normal individual [24,26]. The total number of left-to-right saccades across the lines reduced from 480, which is very abnormal and child-like motorically, to 74, which is adult-like and normal. This represents nearly a 650% improvement, which was the largest we found in any of the individuals trained and tested with mTBI. This suggests a strong motor learning effect [29]. In addition to these improvements, the reading rate (words/minute, wpm) increased from 243 to 308, a 27% increase. The improvements in this patient, and the others, persisted over the six-month follow-up period [20].

## 4. Program Areas for Future Development

There are several areas to consider for future development and advancement of the aforementioned oculomotor training program (COR), as well as the related conceptual ideas. First, the inclusion of an automated stimulus system to alter the vergence and/or accommodative demand(s) over a wide range of values would make the system better controlled, more versatile, more efficient, and less cumbersome. Currently, this function is performed manually either by the patient or doctor. Unfortunately, performing such changes manually, especially if done by the patient, makes perfect optical alignment of the lenses/prisms with the eye somewhat difficult. Second, the inclusion of a video-based system to measure eye movements and accommodation objectively, as well as to monitor the pupil, during the actual training process would advance the system to a new level. This would allow for careful on-line monitoring of these oculomotor systems during the actual testing, as well as for subsequent off-line use incorporating sophisticated, quantitative data analysis and graphical depiction of the system(s) responsitivity over the time of testing, as correlated with the stimulus changes. Thus, one could frequently assess changes and progress in the tested oculomotor system objectively over the period of therapy. This analysis could be performed either manually by the experimenter/clinician, or more preferably by automated computer means with graphical display. Third, oculomotor-based, auditory feedback could be added, so that the patient could “hear” their abnormal eye movements to enhance motor learning [30]. Lastly, the broad aspects and versatility of the COR program allow for its use over a wide range of diagnostic groups manifesting abnormal oculomotor control, in addition to those with TBI, such as that in found in patients with low vision, amblyopia, strabismus, nystagmus, and convergence insufficiency, among others.

## 5. Conclusions

The COR program represents an important step in the successful oculomotor rehabilitation of individuals with TBI. Improvements in oculomotor control were also transferred to reading, a critical activity of daily living (ADL) both vocationally and avocationally. SHAM training showed no effect [26]. With further advancements, especially increased automation and advanced computer programming, the COR program has the potential to be available to anyone, at any time, that has access to a computer or related hand-held technology.

## Figures and Tables

**Table 1:** (**a**): Stimuli for version training protocol [26]; (**b**): Training protocol for vergence and accommodation [26].

**(a)**			
**Stimulus**	**Stimulus Parameter**	**Training Period Duration (s)**	**Total Training Duration (s)**
Fixation	Central (midline)	60	300
	Left (10 degrees)	60	
	Right (10 degrees)	60	
	Up (10 degrees)	60	
	Down (10 degrees)	60	
Predictable Saccades	Horizontal (±5 degrees)	75	300
	Horizontal (±10 degrees)	75	
	Vertical (±5 degrees)	75	
	Vertical (±10 degrees)	75	
Simulated Reading (repeated twice)	Full-screen	75	300
	Single-line	75	
	Full-screen	75	
	Single-line	75	
**(b)**			
**Stimulus**	**Stimulus Parameter**	**Training Period Duration (min)**	**Total Training Duration (min)**
Vergence	Step amplitude (BO/BI *)	7	15
	Step facility (BO/BI *)	5	
	Ramp	3	
Accommodation	Step amplitude right eye lenses	5	15
	Step amplitude left eye lenses	5	
	Step facility	5	

* BO/BI = base-out prism/base-in prism.
